# The Synergistic Effect of Valsartan and LAF237 [(S)-1-[(3-Hydroxy-1-Adamantyl)Ammo]acetyl-2-Cyanopyrrolidine] on Vascular Oxidative Stress and Inflammation in Type 2 Diabetic Mice

**DOI:** 10.1155/2012/146194

**Published:** 2012-03-15

**Authors:** Min Shen, Dongdong Sun, Weijie Li, Bing Liu, Shenxu Wang, Zheng Zhang, Feng Cao

**Affiliations:** Department of Cardiology, Xijing Hospital, Fourth Military Medical University, Xi'an, Shaanxi 710032, China

## Abstract

*Aim*. To investigate the combination effects and mechanisms of valsartan (angiotensin II type 1 receptor blocker) and LAF237 (DPP-IV inhibitor) on prevention against oxidative stress and inflammation injury in db/db mice aorta. *Methods*. Db/db mice (*n* = 40) were randomized to receive valsartan, LAF237, valsartan plus LAF237, or saline. Oxidative stress and inflammatory reaction in diabetic mice aorta were examined. *Results*. Valsartan or LAF237 pretreatment significantly increased plasma GLP-1 expression, reduced apoptosis of endothelial cells isolated from diabetic mice aorta. The expression of NAD(P)H oxidase subunits also significantly decreased resulting in decreased superoxide production and ICAM-1 (fold change: valsartan : 7.5 ± 0.7, *P* < 0.05; LAF237: 10.2 ± 1.7, *P* < 0.05), VCAM-1 (fold change: valsartan : 5.2 ± 1.2, *P* < 0.05; LAF237: 4.8 ± 0.6, *P* < 0.05), and MCP-1 (fold change: valsartan: 3.2 ± 0.6, LAF237: 4.7 ± 0.8; *P* < 0.05) expression. Moreover, the combination treatment with valsartan and LAF237 resulted in a more significant increase of GLP-1 expression. The decrease of the vascular oxidative stress and inflammation reaction was also higher than monotherapy with valsartan or LAF237. *Conclusion*. These data indicated that combination treatment with LAF237 and valsartan acts in a synergistic manner on vascular oxidative stress and inflammation in type 2 diabetic mice.

## 1. Introduction

Diabetes mellitus is responsible for a host of vascular deterioration such as heart disease, nephropathy, and retinopathy. The key roles that hyperglycemia, inflammation, and oxidative stress play in the progression of diabetes vascular complications are well recognized. Reactive oxygen species (ROS), in particular superoxide anion (O_2_^•^−^^), have been shown to initiate several processes involved in diabetes vascular complications, resulting in increased endothelial cells apoptosis and increased expression of adhesion molecules which include intracellular adhesion molecule (ICAM)-1, vascular cell adhesion molecule (VCAM)-1, and monocyte/macrophage chemotactic protein-1 (MCP-1).

Glucagon-like peptide-1 (7–36) amide (GLP-1) is a gut hormone released postprandially that increases glucose-stimulated insulin secretion, suppresses glucagon secretion, and delays gastric emptying [1]. GLP-1 receptors were expressed in the human pancreas, lung, brain, stomach, kidney, and heart [2, 3]. It has been shown that GLP-1 could protect against endothelial dysfunction in type 2 diabetic patients with coronary heart disease [2, 4, 5]. However, GLP-1 was not employed for therapeutic use since it is rapidly cleared from the circulation by the enzyme dipeptidyl peptidase-IV (DPP-IV). Thus, DPP-IV inhibitor could serve as a promising potent therapeutic target for diabetic vascular deterioration. It has been well recognized that angiotensin II receptor blockers (ARBs) could alleviate endothelial dysfunction in diabetic patients. However, whether DPP-IV inhibitor (LAF237) and valsartan have synergistic effect of on vascular oxidative stress and inflammation in type 2 diabetic mice is still not well understood.

 Therefore, the aims of the present study were (1) to identify the expression of GLP-1 receptor in the diabetic mice aorta and the effects of valsartan (angiotensin II type 1 receptor blocker) and LAF237 (DPP-IV inhibitor) on plasma GLP-1 expression; (2) to investigate the synergistic effect of valsartan and LAF237 on vascular oxidative stress and inflammation in type 2 diabetic mice.

## 2. Materials and Methods

### 2.1. Animal Procedures

The experiments were performed in adherence with The Code of Ethics of the World Medical Association (Declaration of Helsinki) and were approved by the Fourth Military Medical University Committee on Animal Care. Four-week old genetically diabetic C57BL/KSJ db/db mice and their age-matched nondiabetic littermates C57/KSJ m/db were obtained from the Laboratory Animal Services Center of the Fourth Military Medical University. Db/db mice were randomly divided into four groups: (1) valsartan-treated (10 mg·kg^−1^·day^−1^) group (*n* = 10) (val), (2) LAF237-treated (1 mg·kg^−1^·day^−1^) group (*n* = 10) (LAF), (3) valsartan- and LAF237-combo-treated (valsartan 10 mg·kg^−1^·day^−1^ + LAF 1 mg·kg^−1^·day^−1^) group (*n* = 10) (combo), (4) vehicle-treated db/db group (*n* = 10) (db/db), and (5) one group of m+/db mice (*n* = 10) was used as negative control (m+/db). Mice were treated with valsartan and LAF237 for 8 weeks. Blood from the tip of the tail was used to measure blood glucose concentration by using a reflectance meter (Accu-Chek, Roche Diagnostics GmbH, Mannheim, Germany). Plasma triglycerides, cholesterol, LDL, and HDL were measured spectrophotometrically by an automated system (Dade Behring RxL Max). The mice were fasted overnight before the last day of valsartan or LAF237 treatment. One hour after the last dose of valsartan or LAF237 was given, an oral glucose tolerance test (2 g/kg) was performed. The plasma active GLP-1 was measured with a commercial assay (Linco Research, St. Charles, MO) 5 min after glucose loading.

### 2.2. Immunochemistry Staining of GLP-1 Receptor

After pretreatment with valsartan and LAF237 for 8 weeks, aortas were isolated from the mice in each group and placed immediately in ice-cold phosphate-buffered saline (PBS). For immunoperoxidase staining, the ABC staining technique was applied to illustrate the specific localization of GLP-1 receptor in aorta. Aortas were cut into 4 mm segments. The aortic segments were fixed in 4% paraformaldehyde and embedded in paraffin. Sections of 5 *μ*m thickness were deparaffinized and rehydrated through graded alcohols to water. Endogenous peroxidase was blocked with 3% hydrogen peroxide for 20 min. After antigen retriever, the sections were incubated with 6% normal donkey serum for 1 hour at room temperature to block nonspecific antibody binding. The sections were then incubated with anti-GLP-1 receptor rabbit polyclonal antibody (1 : 1000) at 4°C overnight. After three times washing with PBS, the primary antibody was detected with the biotinylated secondary antibody from the Vectastain Elite ABC Kit (rabbit IgG) (Vector Laboratories) at room temperature for 1 h. After another three washes with PBS, the sections were incubated with the Vectastain Elite ABC Reagent for 1 h at room temperature. The sections were than washed three times in PBS and incubated in peroxidase substrate kit DAB (Vector Laboratories) for 7 min for brown color development. Omission of primary antibodies was used as negative controls. The positive brown staining was detected under a light microscope equipped with a DFC490 digital camera (Leica Microsystems).

### 2.3. Determination of Endothelial Cells Apoptosis

After pretreatment with valsartan and LAF237 for 8 weeks, aorta intima was isolated from the mice in each group. Endothelial cells apoptosis was analyzed by commercially available *in situ* cell death detection ELISAplus assay (Roche Diagnostics) according to manufacturer's instructions. The assay was repeated for three times to confirm the result. Caspase-3 activity was measured with the ApoAlert Caspase-3 Assay Plate (Clontech, Mountain View, CA) according to the manufacturer's protocols [6]. Values are expressed as arbitrary units of caspase-3/*μ*g heart tissue.

### 2.4. Real-Time PCR Analysis

The entire aortas from five groups were homogenized, and total RNA was isolated using Trizol reagent (Invitrogen) according to the manufacturer's instructions. The RNA yield was measured by absorbance at 260 nm. The first strand cDNA was synthesized by Superscript II reverse transcriptase with oligo(dT)_12–18_ primer from 1 *μ*g of total RNA. To ensure that the primers were specific and efficient, a number of tests were performed. Quantitative RT-PCR was performed in duplicate using the following primer sequences: gp91phox, sense 5′-TGC CCA GTA CCA AAG TTC G-3′, and antisense 5′-TGT CCC ACC TCC ATC TTG A-3′; p22phox, sense 5′-GTC ATG GGG CAG ATC GAG T-3′, and antisense 5′-AGC ACA CCT GCA GCG ATA G-3′; VCAM-1, sense 5′-CGA GGC TGG AAT TAG CAG A-3′, and antisense 5′-CAT GTT TCG GGC ACA TTT C-3′; ICAM-1, sense 5′-GAC CAC GGA GCC AAT TTC T-3′, and antisense 5′-GTC AGG GGT GTC GAG CTT T-3′; MCP-1, sense 5′-AGT TAA CGC CCC ACT CAC C-3′, and antisense 5′-TGG TTC CGA TCC AGG TTT T-3′; **β**-actin, sense 5′-CTG TAT TCC CCT CCA TCG T-3′, and antisense 5′-GCC ATG TTC AAT GGG GTA CT-3′.

The amplification was carried out in 25 *μ*L of reaction mixture containing 12.5 *μ*L of SYBR Green Supermix (Bio-Rad), 1 *μ*L of the first strand cDNA products, and 3 *μ*M each of the sense and antisense primers. For each set of primers, a no-template control was included. The reactions were carried out on a 96-well plate, and a PCR amplification protocol was followed (95°C for 5 min and 45 cycles of amplification at 95°C for 30 s, 58°C for 30 s, and 72°C for 30 s) using an iCycler machine (Bio-Rad). Postamplification melting curve analysis was performed to show a single amplification product without contamination. Electrophoresis analysis on a 1.5% agarose gel was also carried out for quality control. The fold relative to **β**-actin for the mRNA of interest was calculated using the 2^−  ΔΔT^ method.

cDNA of m+/db mice aorta was synthesized as described above. RT-PCR was performed in duplicate to detect mRNA expression of GLP-1 receptor in aorta, using GLP-1 receptor primer sequence, sense 5′-CTC TTT GCT ATC GGC GTC A-3′ and antisense 5′-AGC CCT GGA AGG AAG TGA A-3′. PCR was performed using the following thermal cycle: 5 minutes at 94°C, followed by 35 cycles of 30 seconds at 94°C, 30 seconds at 58°C, and 1 minute at 72°C. cDNA of m+/db mice pancreas was used as positive control. The PCR products were demonstrated by electrophoresis on 1.5% agarose gels.

### 2.5. Localization and Quantification of Superoxide Anion

To assess in situ O_2_^•^−^^ production, dihydroethidium (DHE) fluorescence of aortic tissue sections was used. Aortae were prepared as described above, and 4 mm segments were embedded in a resin (Tissue-Tek O.C.T. 4583 Compound) and frozen, and stored at −80°C. Samples were sectioned on a Leica cryostat (20 *μ*m) and placed on glass slides and fixed with 4% paraformaldehyde for 10 min at room temperature. DMSO containing 2 *μ*mol/L DHE was topically applied to each tissue section, and sections were incubated in a dark, humidified chamber at 37°C for 30 minutes. Omission of DHE was used as negative controls. DHE (red) and autofluorescence (green) were detected with a fluorescent microscope equipped with a DFC490 digital camera (Leica Microsystems). Computer-based analysis was performed using Olympus Fluoview ver 1.5 software (Olympus). Data were expressed as % fluorescence/mm^2^.

### 2.6. Immunofluorescence Staining of Monocyte Chemotactic Protein-1 (MCP-1)

 Cryosections of aorta were used for MCP-1 immunofluorescence staining. Sections were prepared as described above and incubated with 6% normal donkey serum for 1 hour at room temperature to block nonspecific antibody binding. The sections were then incubated with anti-MCP-1 rabbit polyclonal antibody (1 : 100) (Santa Cruz Biotechnology) at 4°C overnight. After three times washes with PBS, the primary antibody was detected with rhodamine-conjugated donkey anti-rabbit IgG (1 : 100) (Jackson ImmunoResearch) for 1 hour at room temperature. Omission of primary antibody was used as negative controls. MCP-1 (red) immunolabeling was detected with a fluorescent microscope equipped with a DFC490 digital camera (Leica Microsystems).

### 2.7. Western Blot Assay

Protein was isolated from mice aorta with Trizol reagent (Invitrogen, Carlsbad, CA) and standard Invitrogen protocols as previously described [6, 7]. In brief, the Bradford assay (Bio-Rad Laboratories, Hercules, CA) was used to quantify protein concentrations. Protein was then used for western blotting with primary antibodies against GLP-1R. All of the antibodies were purchased from Santa Cruz Biotechnology (Santa Cruz, Calif). The blots were visualized with a chemiluminescence system (Amersham Bioscience, Buchinghamshire, UK). The signals were quantified by densitometry.

### 2.8. Statistical Analysis

Results of quantitative studies were expressed as means ± SD. Each data point represents the average of 5 to 6 experiments. Multiple comparisons between groups were performed by using an analysis of variance (ANOVA) followed by Turkey's post hoc test. Statistical significance was accepted for *P* value less than 0.05.

## 3. Results

### 3.1. Basic Parameters

Blood glucose was significantly decreased in valsartan, LAF237, and combo treatment group compared with vehicle-treated db/db group. The combination treatment with valsartan and LAF237 for 8 weeks significantly decreased triglycerides, total cholesterol, and LDL and increased plasma HDL compared with vehicle-treated db/db group (Table 1).

### 3.2. Detection of GLP-1 Receptor (GLP-1R) and the Effects of Valsartan and LAF237 on Plasma GLP-1 Expression

**Figure 1:**
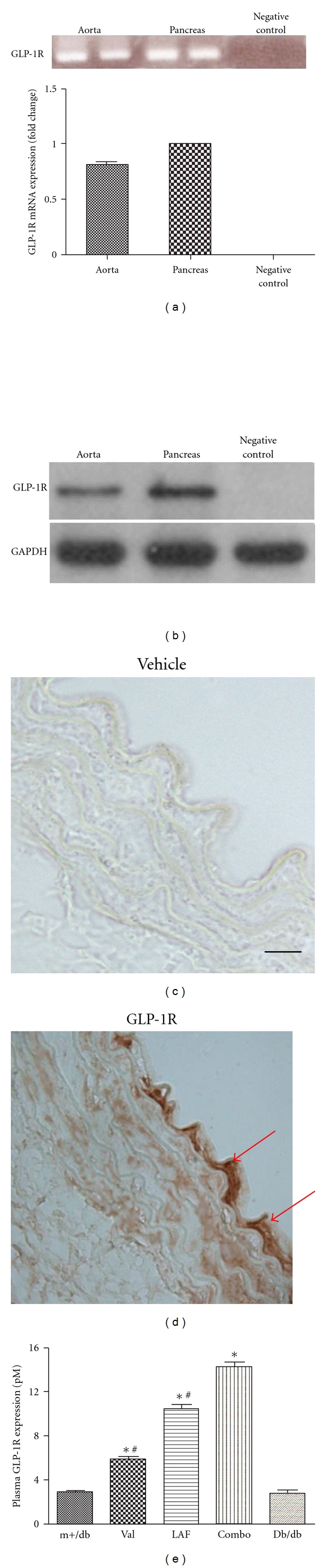
Detection of GLP-1R and the effects of valsartan and LAF237 on plasma GLP-1 expression. GLP-1R mRNA expression was measured by RT-PCR. Pancreas was utilized as positive control (a). Western blot analysis showed that GLP-1R was expressed in db/db mice aorta (b). Negative control of GLP-1 receptor immunostaining (c). GLP-1 receptor was expressed on endothelium of diabetic mice aorta showed in brown (bar, 50 *μ*m) (d). The plasma GLP-1 expression was increased by valsartan or LAF237 pretreatment. The combination treatment with valsartan and LAF237 showed more effective increase than that of single treated group (e). The columns and errors bars represent means and SD. *P < 0.05 versus db/db group, ^#^ *P* < 0.05 versus combined treatment group.

 GLP-1 receptor expression in diabetic mice aorta was detected by real-time PCR (RT-PCR), western blot, and immunohistochemistry staining. Figures 1(a) illustrated the RT-PCR products generated from RNA extracted from aorta and pancreas. GLP-1 receptor mRNA was detectable both in db/db mice aorta and pancreas. Western blot analysis also showed that GLP-1R was expressed on db/db mice aorta (Figures 1(b)). Immunohistochemistry was used to further determine that GLP-1 receptor was expressed on diabetic mice aorta (Figures 1(c), 1(d)). Valsartan (5.8 ± 0.6 versus  2.7 ± 0.8, *P* < 0.05) or LAF237 (10.4 ± 1.0 versus 2.7 ± 0.8, *P* < 0.05) administration significantly increased plasma GLP-1 expression compared with the db/db group. Furthermore, combination treatment with valsartan and LAF237 showed more effective increase than that of single-treated group (combo: 14.2 ± 1.1  versus  val: 5.8 ± 0.6 versus  LAF: 10.4 ± 1.0; *P* < 0.05) (Figures 1(e)). 

### 3.3. Antiapoptotic Effects of Valsartan and LAF237 on Endothelial Cells of Diabetic Mice Aorta

**Figure 2:**
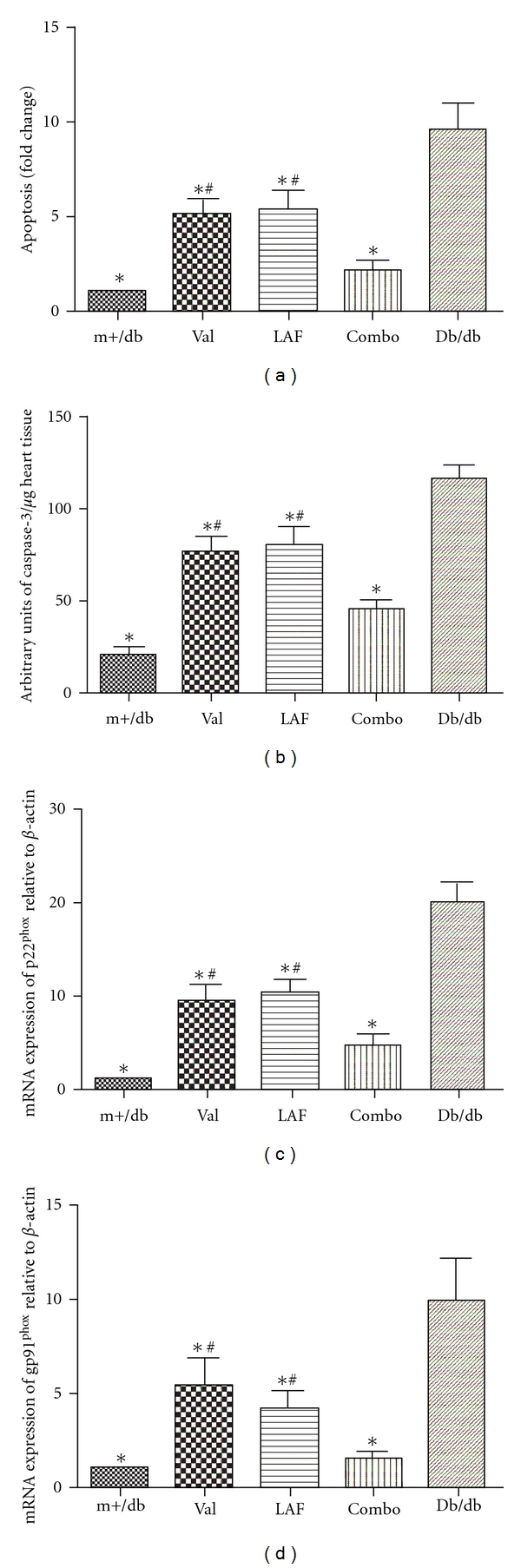
Effects of valsartan and LAF237 on apoptosis and NA(D)PH oxidase subunits of endothelial cells of db/db mice aorta. DNA fragmentation of each group was detected by ELISA. Valsartan and LAF237 pretreatment significantly reduced endothelial cells apoptosis. The combined treatment could further reduce endothelial cells apoptosis (a). Valsartan and LAF237 significantly decreased caspase-3 activity compared with the db/db group (b). The expression of p22^phox^ and gp91^phox^  subunits was significantly decreased compared with db/db mice after treating with valsartan or LAF237. The combo-treated group showed more effectively decrease than that of single treated group (c, d). The columns and errors bars represent means and SD. *P < 0.05 versus db/db group, ^#^ *P* < 0.05 versus combined treatment group.

DNA fragmentation in diabetic mice aorta was detected by *in situ* cell death ELISA (Figures 2(a)). The degree of DNA fragmentation was about 9.6 times higher in db/db group compared with m+/db nondiabetic mice. The level of DNA fragmentation was significantly reduced with the pretreatment of valsartan (−46%; *P* < 0.05) or LAF237 (−45%; *P* < 0.05). Moreover, the combined treatment could further reduce endothelial cells apoptosis (−78%; *P* < 0.05).

We also determined levels of caspase-3 activity and found that valsartan (75.7 ± 8.4 versus   115.0 ± 8.1, *P* < 0.05) and LAF237 (78.8 ± 10.9 versus  115.0 ± 8.1, *P* < 0.05) statistically decreased caspase-3 enzymatic activity compared with the db/db group. The combination treatment significantly reduced caspase-3 activity compared with valsartan (44.6 ± 5.3   versus 75.7 ± 8.4, *P* < 0.05) or LAF237 (44.6 ± 5.3 versus 78.8 ± 10.9, *P* < 0.05) single treatment (Figures 2(b)).

### 3.4. Effects of Valsartan and LAF237 on NA(D)PH Oxidase Subunits of Diabetic Mice Aorta

The mRNA expression of the  p22^phox  ^ and gp91^phox^ subunits of NA(D)PH oxidase was measured by real-time PCR analyses. The expression of p22^phox  ^  and gp91^phox^ subunits of NAD(P)H oxidase was significantly increased in db/db mice comparing with m+/db mice (fold change: p22^phox  ^ (9.4 ± 1.6) and gp91^phox^ (5.4 ± 1.5); *P* < 0.05). The expression of p22^phox  ^ and gp91^phox^ subunits was significantly decreased compared with db/db mice after treating with valsartan (p22^phox  ^ −53% and gp91^phox^ −46%; *P* < 0.05) or LAF237 (p22^phox  ^ −48% and gp91^phox^ −57%; *P* < 0.05). The combo-treated group showed more effective decrease than that of single-treated group (p22^phox  ^ −77% and gp91^phox^ −83%; *P* < 0.05) (Figures 2(c), 2(d)).

### 3.5. In Situ Measurement of *O*_2_^•^−^^ Production in Aortic Segments

**Figure 3:**
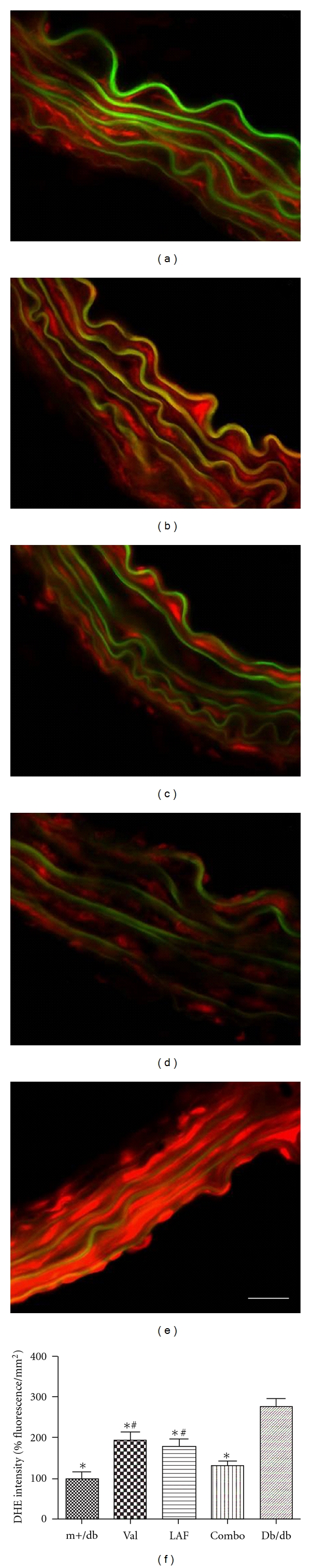
*In situ* measurement of O_2_^•^−^^ production in aortic segments. ROS generation within the aorta determined by dihydroethidium (DHE) fluorescence (×630 magnification). Fluorescence photomicrographs at identical settings of sections of thoracic aorta labeled with DHE. Positive staining was shown in red, and green part indicated auto-fluorescence. (a) m+/db mouse aorta; (b) valsartan-treated mouse aorta; (c) LAF237-treated mouse aorta; (d) combo-treated mouse aorta; (e) nontreated db/db mouse aorta (bar, 10 *μ*m). (f) Quantification of the fluorescence signal showed that the fluorescence intensity was significantly decreased in valsartan or LAF237 pretreatment group compared with m+/db group. The columns and errors bars represent means and SD. *P < 0.05 versus db/db group, ^#^ *P* < 0.05 versus combined treatment group.

Dihydroethidium (DHE, Invitrogen), an oxidative fluorescent probe, was used to detect *in situ* oxidative stress. Morphologic analysis revealed intracellular formation of O_2_^•^−^^ in vessel area containing endothelial cells and medial smooth muscle cells (SMCs) (Figure 3). Quantification of the fluorescent signal showed a significant increase of fluorescence intensity in the aorta of db/db mice compared with m+/db group (fold change: 2.2 ± 0.4; *P* < 0.05). After treated with valsartan or LAF237, the fluorescence intensity was significantly decreased (fold change: 1.6 ± 0.3 and 1.4 ± 0.3; *P* < 0.05) compared with m+/db group. Moreover, the combo-treated group showed more significant decrease than that of single treated group (fold change: 1.1 ± 0.2; *P* < 0.05) (Figure 3).

### 3.6. Effects of Valsartan and LAF237 on Adhesion Molecules in Diabetic Mice Aorta

**Figure 4:**
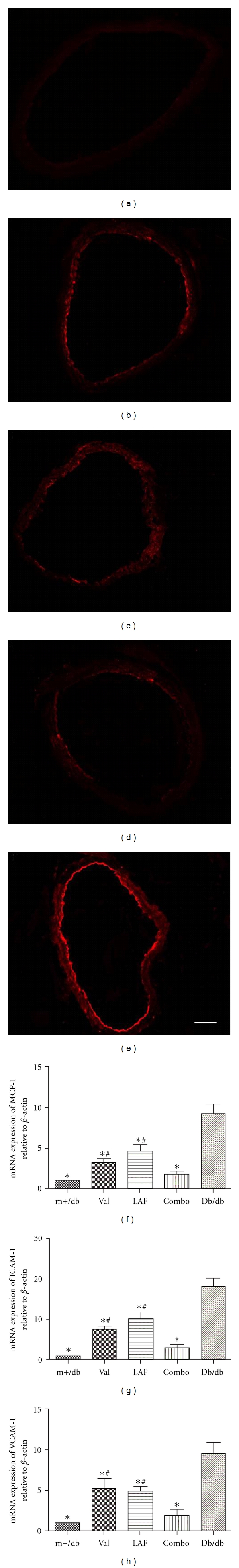
Effects of valsartan and LAF237 on adhesion molecules in db/db mice aorta. Representative images of MCP-1 expression were shown in Figures 4(a)–4(e). (a) m+/db mice aorta; (b) valsartan-treated mice aorta; (c) LAF237-treated mice aorta; (d) combo-treated mice aorta; (e) nontreated db/db mouse aorta. The intensity of fluorescence was reduced in valsartan and LAF237-treated group (bar, 25 *μ*m). The mRNA expression of MCP-1(f), ICAM-1(g), and VCAM-1(h) was evaluated by real-time PCR. The columns and errors bars represent means and SD. *P < 0.05 versus db/db group, ^#^ *P* < 0.05 versus combined treatment group.

To demonstrate MCP-1 expression on the aorta endothelium, immunofluorescence staining was performed. Figures 4(a)–4(e) illustrated that MCP-1 was located on db/db aorta endothelium. The intensity of fluorescence was reduced in valsartan- and LAF237-treated group. Real-time PCR was used to further confirm the expression of MCP-1. The mRNA expression of MCP-1 was increased in db/db group compared with m+/db nondiabetic group (fold change: 9.3 ± 1.1, *P* < 0.05). After treated with valsartan or LAF237, the expression of MCP-1 was significantly decreased (fold change: 3.2 ± 0.6 and 4.7 ± 0.8; *P* < 0.05) compared with m+/db group. The combination-treated group further decreased MCP-1 expression (fold change: 1.8 ± 0.4, *P* < 0.05) (Figures 4(f)).

The mRNA expression of ICAM-1 (fold change: 18.3 ± 1.9, *P* < 0.05) and VCAM-1 (fold change: 9.6 ± 1.2, *P* < 0.05) was also significantly increased in db/db group compared with m+/db group. Valsartan and LAF237 pretreatment significantly decreased ICAM-1 (valsartan: 7.5 ± 0.7, *P* < 0.05; LAF237: 10.2 ± 1.7, *P* < 0.05) and VCAM-1 (valsartan: 5.2 ± 1.2, *P* < 0.05; LAF237: 4.8 ± 0.6, *P* < 0.05) expression. The combo-treated group showed more significant decrease compared with single-treated group (Figures 4(g), 4(h)).

## 4. Discussion

Glucagon-like peptide-1 (GLP-1), the most potent insulinotropic hormone known, increases glucose-stimulated insulin secretion, suppresses glucagon secretion and delays gastric emptying [8, 9]. However, therapeutic use of GLP-1 itself is severely compromised by its lack of oral activity and rapid degradation by plasma dipeptidyl peptidase IV (DPP-IV). Compelling studies have shown that inhibition of DPP-IV was effective in improving glucose tolerance in diabetic patients in human clinical trials since DPP-IV inhibition could extend the duration of GLP-1 action and prolong its beneficial effects [10, 11]. Therefore, DPP-IV inhibitor (LAF237) offers a new strategy for treating type 2 diabetes. Several studies have explored the expression of GLP-1 receptor in pancreatic islet cells, gastrointestinal tract, heart, lung, and so forth [12]. In the present study, we also demonstrated that GLP-1 receptor is expressed on diabetic mice aorta as evidenced by RT-PCR, western blot, and immunohistochemistry staining.

Endothelial dysfunction can be improved by inhibition of rennin-angiotensin system using ACEI or ARB, not only in patients with coronary artery disease, hypertension, and chronic heart failure, but also in patients with diabetes [13]. Angiotensin II could interact with oxygen radicals and stimulates the generation of oxygen radicals by increasing the expression of the nicotinamide adenine dinucleotide phosphate via activation of the AT1 receptor. Previous studies indicated that LAF237 (inhibitor of dipeptidyl peptidase IV) and valsartan (antagonist of the angiotensin II type 1 receptor) have significant beneficial additive effects on pancreatic *β*-cell structure and function compared with their respective monotherapeutic effects [14]. The aim of the present study is to investigate the combination effects of valsartan and LAF237 on vascular oxidative stress and inflammation in db/db mice.

In the present study, we demonstrated that both of valsartan and LAF237 increased plasma GLP-1 expression compared with the vehicle-treated group. Furthermore, combination treatment with valsartan and LAF237 showed more effective increase than that of single-treated group. We also explored the antiapoptotic effects of valsartan and LAF237 on endothelial cells of diabetic mice aorta. Both of them reduced endothelial cells apoptosis. Moreover, the combination therapy of valsartan and LAF237 induced a significantly lower apoptosis rate compared with monotherapy.

Diabetes is associated with an increased risk for vascular deterioration. Endothelial cells regulate basal vascular tone and vascular reactivity with the release of a variety of contracting and relaxing factors. It has been shown that diabetes can cause endothelial cell damage by several mechanisms, including apoptosis and increased oxidative stress. Oxidative stress implies a loss of redox homeostasis with an excess of ROS production by singular process of oxidation. ROS include free radicals such as superoxide anion, peroxynitrite, hydroxyl, and H_2_O_2_. Loss of the modulatory role of the endothelium may play a key role in the development of diabetic vascular dysfunction [15, 16].

Here, we used dihydroethidium staining to localize *in situ *O_2_^•^−^^ production. DHE is a fluorescent dye, which specifically reacts with intracellular O_2_^•^−^^and is converted to the red fluorescent compound ethidium. DHE red fluorescence was localized mainly in endothelial cells and smooth muscle layers according to the production profile of O_2_^•^−^^ within the arteries. In the present study, we found that fluorescence intensity in the aorta isolated from nontreated db/db mouse was about 2 times higher than that of the m+/db mice, indicating an increase in O_2_^•^−^^formation. After treated with valsartan or LAF237 for 8 weeks, O_2_^•^−^^production was significantly decreased. Meanwhile, combo-treated mouse showed further more reduced O_2_^•^−^^formation.

NAD(P)H oxidase, which catalyzes the NADPH-dependent reduction of oxygen to form superoxide, is one of the most important sources of superoxide production in vascular tissue. Vascular NAD(P)H oxidase is a multimeric protein complex that consists of plasma membrane-bound subunits (gp91phox/Nox2 and p22phox) and cytosolic subunits (p40phox, p47phox, p67phox, and small GTPase Rac1). A strong correlation between p22^phox^ expression and superoxide production in human coronary artery disease (CAD) has been reported [17]. NAD(P)H oxidase activity in CAD was associated with elevated expression levels of p22^phox^ and gp91^phox^ [18]. Roberts et al. demonstrated that NAD(P)H oxidases were strongly involved in impaired vascular function in the thoracic aorta of rats fed a diet enriched with sugar and fat [19].

In the present study, we have explored the role of NAD(P)H oxidase gp91^phox^ and  p22^phox^ subunits as modulators of ROS production in diabetic mice. The mRNA expression of gp91^phox^ and p22^phox^ subunits of NAD(P)H oxidase was significantly increased in the diabetic mice aortic. Valsartan and LAF237 pretreatment significantly decreased NAD(P)H oxidase gp91^phox^ and p22^phox^ subunits expression. The combo treatment with valsartan and LAF237 could further reduce the mRNA expression of the above units, indicating a synergistic effect of valsartan and LAF237 on vascular oxidative stress in diabetic mice.

During the development of diabetes, a number of biochemical and mechanical factors converge on the endothelium, resulting in endothelial dysfunction and vascular inflammation. The inflammatory site in endothelial dysfunction may be where the process of inflammation in type 2 diabetes begins. An increased adhesion of leukocytes (especially monocytes) to the endothelium followed by transmigration into the subendothelial space is a crucial early event in the pathogenesis of atherosclerosis and certain inflammation disorders. It has been well recognized that increased levels of ROS activate signal transduction processes leading to expression of proinflammatory cytokines/chemokines such as TNF-*α*, IL-1*β*, IL-6, MCP-1, and IL-8, as well as adhesion molecules such as VCAM-1 and ICAM-1 in endothelial cells, microglia, and astrocytes [20]. Hyperglycemia could also increase the expression of ICAM-1, VCAM-1, and MCP-1 release [13, 21, 22].

 The present data demonstrated that mRNA expression of ICAM-1 and VCAM-1 in db/db mouse was much higher than in m+/db mouse, after treated with valsartan or LAF237, and the expression of these adhesion molecules was decreased. Furthermore, the combo treatment induced a more significant decrease compared with the single-treated group. In db/db mouse, MCP-1 release was significantly increased as evidenced by staining with monoclonal antibody against MCP-1 and real-time PCR. The combo treatment with valsartan and LAF237 also had a more significant decrease of MCP-1 compared with single treatment using either valsartan or LAF237.

 In conclusion, these results indicated that oxidative stress and inflammatory reaction were evident in db/db mice aorta. NADPH oxidase played a pivotal role in ROS production, resulting in increased expression of adhesion molecules such as VCAM-1, ICAM-1, and MCP-1. Valsartan or LAF237 significantly reduced oxidative stress and inflammatory reaction in diabetic mice aorta as evidenced by reduced apoptosis and reduced expression of NAD(P)H oxidase submits, ROS production, ICAM-1, VCAM-1, and MCP-1 expression. Furthermore, LAF237 and valsartan act in a synergistic manner on vascular oxidative stress and inflammation in type 2 diabetic mice. The possible mechanism responsible for the effects of valsartan and LAF237 is associated, at least in part, with the upregulation of plasma GLP-1 expression.

## 5. Study Limitations

The present study did not reveal the exact molecular pathways of valsartan and LAF237 act on. The detailed mechanism of how valsartan upregulates plasma GLP-1 expression and the mechanism of valsartan and LAF237 on the diabetic complication remains to be clarified.

##  Conflict of Interests 

 Nothing to declare.

##  Authors' Contribution 

Min Shen and Dongdong Sun Contributed equally to this work.

## Figures and Tables

**Table 1:** Basic parameters in db/db mice and their controls.

Basic parameters	m+/db (*n* = 10)	Valsartan (*n* = 10)	LAF237 (*n* = 10)	Combo (*n* = 10)	db/db (*n* = 10)
Body mass (g)					
4w	17.5 ± 1.9	26.3 ± 2.1	25.6 ± 2.8	24.1 ± 2.2	25.8 ± 3.4
12w	27.5 ± 2.9^∗#^	41.2 ± 4.0^#^	35.3 ± 3.7*	36.4 ± 3.9*	40.8 ± 4.3
Glucose (mmol/L)					
4w	6.3 ± 1.2^∗#^	8.7 ± 1.1	8.2 ± 1.4	8.5 ± 0.8	8.1 ± 1.2
12w	8.2 ± 1.4^∗#^	22.4 ± 2.7^#^	17.5 ± 2.4*	17.8 ± 2.2*	25.3 ± 3.3
Triglycerides (mmol/L)					
4w	0.65 ± 0.07^∗#^	1.23 ± 0.14	1.21 ± 0.18	1.24 ± 0.16	1.22 ± 0.23
12w	0.70 ± 0.05^∗#^	1.73 ± 0.20	1.65 ± 0.18*	1.62 ± 0.14*	1.85 ± 0.29
Cholesterol (mmol/L)					
4w	2.37 ± 0.26^∗#^	3.70 ± 0.32	3.73 ± 0.45	3.68 ± 0.34	3.71 ± 0.41
12w	2.42 ± 0.18^∗#^	3.46 ± 0.32^#^	3.23 ± 0.24*	3.15 ± 0.28*	3.52 ± 0.38
LDL (mmol/L)					
4w	0.96 ± 0.03^∗#^	1.53 ± 0.12	1.55 ± 0.17	1.56 ± 0.15	1.54 ± 0.22
12w	0.92 ± 0.04^∗#^	1.38 ± 0.11*	1.42 ± 0.16*	1.37 ± 0.16*	1.63 ± 0.21
HDL (mmol/L)					
4w	1.17 ± 0.15^∗#^	1.55 ± 0.14	1.53 ± 0.16	1.56 ± 0.18	1.58 ± 0.17
12w	1.20 ± 0.16^∗#^	1.69 ± 0.18^∗#^	1.83 ± 0.25	1.98 ± 0.27*	1.73 ± 0.14

Values are presented as mean ± SD.

*P < 0.05 versus db/db group, ^#^ *P* < 0.05 versus combined treatment group.
